# Clinical efficacy, safety, and success factors of botulinum toxin type A in the treatment of acute acquired comitant esotropia

**DOI:** 10.1007/s10384-026-01331-6

**Published:** 2026-02-12

**Authors:** Makiko Ui, Toshiaki Goseki, Ayumi Watanabe, Kinue Fueki, Yuko Komiya, Risako Hasunuma, Noriko Tsutsumi, Hiroki Yoshida

**Affiliations:** 1 CS eye clinic 5F⋅8F Biko-Hongo Building, 3-15-1 Hongo, Bunkyo-ku, Tokyo, Japan; 2 Department of Ophthalmology, International University of Health and Welfare, Atami Hospital, 13-1 Higashi-kaigan-cho, Atami-City, Shizuoka 413-0012 Japan; 3 Department of Ophthalmology, Kitasato University, 1-15-1 Kitasato, Minami-ku, Sagamihara-City, Kanagawa Japan; 4 Data Seed Inc., 5-5-2-103 Mizumoto, Katsushika-ku, Tokyo, Japan

**Keywords:** Acute acquired comitant esotropia (AACE), Botulinum toxin type A (BTXA), Strabismus, Success rate, Success factors

## Abstract

**Purpose:**

Acute acquired comitant esotropia (AACE) is an increasingly reported form of esotropia, possibly linked to the rising use of digital devices. Botulinum toxin type A (BTXA) has emerged as a minimally invasive treatment alternative to surgery, particularly for patients with diplopia. We aimed to investigate the efficacy and safety of BTXA injections in treating AACE while identifying factors contributing to successful treatment outcomes.

**Study design:**

A retrospective, single-center study.

**Methods:**

This study retrospectively evaluated 228 patients with AACE who received BTXA injections at a single center. Treatment outcomes, adverse events, and factors influencing success were analyzed. Successful outcomes were defined as either the absence of diplopia in the primary position or an angle of deviation of ≤10 prism diopters from orthotropia, both evaluated at more than 6 months after the last injection.

**Results:**

The average patient age was 30.9 years, and 52% achieved successful outcomes. Transient adverse events, including blepharoptosis (24%) and hypertropia (16%), were observed. The study found that shorter duration from onset to treatment (P = 0.018) and post-treatment overcorrection (P = 0.0073) were associated with higher success rates. Although some patients required additional treatment or surgery due to recurrence, BTXA was an effective intervention for AACE, with a significant improvement in eye alignment and stereopsis.

**Conclusion:**

Early intervention with BTXA is crucial for successful outcomes and offers a safe, less invasive alternative to traditional strabismus surgery.

## Introduction

Acute acquired comitant esotropia (AACE) is a rare form of esotropia [1]. There has been a notable increase in reported cases recently, likely due to the rising use of digital devices, such as smartphones [2–10]. AACE typically manifests as late-onset esotropia with diplopia in individuals with normal binocular vision [9–11]. Unlike other forms of esotropia that usually appear in early childhood or are associated with significant hyperopic refractive errors or neurological issues, AACE typically occurs in older children and adults. It is characterized by late-onset esotropia with diplopia, consistent esotropia across all gaze directions, minimal or no significant hyperopia, and the absence of organic diseases such as brain tumors [1–9]. Neurological examinations and imaging studies, including computed tomography (CT) or magnetic resonance imaging (MRI), generally return normal results, distinguishing AACE from other causes of acute-onset esotropia.

Recently, botulinum toxin type A (BTXA) injections have emerged as a less invasive alternative to traditional strabismus surgery [12–16]. BTXA temporarily paralyzes the overactive medial rectus muscles, thereby facilitating ocular realignment. Studies demonstrate that BTXA can effectively reduce esotropia and improve binocular function with a relatively low risk of complications. In some cases, the effects of BTXA extend beyond the drug’s active period, leading to long-term alignment. Conversely, it remains unclear which specific cases result in long-term success. Moreover, few large-scale studies report these outcomes.

This study aimed to investigate the efficacy and safety of BTXA injections in treating AACE while also identifying factors that contribute to successful treatment outcomes.

## Materials and methods

### Ethic declarations

This study was approved by the Japanese Medical Association Ethics Committee (approval no. R5-12) and adhered to the ethical standards outlined in the Declaration of Helsinki. The institutional review board granted the study an informed consent waiver and approved the study protocol.

### Patients

This retrospective, single-center study included patients diagnosed with AACE at the CS Eye Clinic who received their first BTXA injection from the same surgeon between November 2019 and June 2023. Medical records from November 2019 to June 2024 were reviewed.

The diagnostic criteria for AACE in this study were partially modified from previous reports [1, 2, 10, 11] and included: (1) onset of diplopia or esotropia after growth, (2) no limitation in ocular motility, (3) absence of organic disease on cranial or orbital CT or MRI, (4) no accommodative component, (5) no history of systemic disease or head trauma, (6) no prior ocular issues such as strabismus or amblyopia, and (7) for patients aged ≥50 years, no disruption of the Lateral rectus-Superior rectus band on orbital MRI suggestive Sagging Eye Syndrome [17, 18]. Patients whose exact onset date could not be determined were included, as, according to previous reports and our clinical experience, AACE frequently develops subacutely or insidiously [4, 19]. Notably, patients who showed reduced esotropia with hyperopic glasses or exhibited convergence spasms were excluded.

### Patient data

The following background information of the patients was obtained from their medical records: sex, age at the first injection, and the period from the onset of diplopia to the first injection. We retrospectively examined the number of BTXA injections, dosage, pre- and post-injection distance eye positions (qualitative and quantitative), near stereopsis, outcomes, and adverse events. Measurements of post-injection distance eye position and near stereopsis were obtained from all available data at 1 month after the first injection and from the final visit in cases with a follow-up period of ≥6 months. In the surgery group shown in the following section, eye position and near stereopsis data were obtained immediately preoperatively. The qualitative distance eye position was assessed using a cover test with a distant target (5 m), whereas the quantitative distance eye position (angle of deviation) was measured using an alternate prism cover test (APCT) at a distance. Near stereopsis was measured using the Stereo Fly test (Stereo Optical Inc.) or the Randot Stereo test (Stereo Optical Inc.).

### BTXA injection procedure and dosage determination

BTXA (Botox^®^, Allergan plc) was injected transconjunctivally into the medial rectus muscle under topical anesthesia with 4% lidocaine eye drops, using electromyographic guidance. As reported in 2019 [20], we encountered several cases where patients showed transient abnormal head posture due to limited ocular motility of the deviating eye after BTXA injection into that eye. Therefore, as a general rule, the injection was administered to the fixing (non-deviating) eye whenever patient consent was obtained. In all cases, the same surgeon measured the pre-injection angle of deviation using APCT. The target dose was determined according to the maximum deviation before reversal (i.e., the largest measured prism diopter (PD)). The initial dose was set based on the degree of deviation as follows:

<12 PD = 1.25 units; 12–<20 PD = 2.0 units; 20–<30 PD = 2.5 units; 30–<45 PD = 4.0 units; and ≥45 PD = 5.0 units.

For subsequent injections, the dose was adjusted according to the clinical response to the previous injection.

### Outcome definitions

Outcomes were evaluated for cases with a follow-up of more than 6 months after the first injection, defined as follows:

- Success: Absence of diplopia in the primary position or an angle of deviation of ≤10 PD from orthotropia, both evaluated at more than 6 months of follow-up after the last injection.
- Improvement: Reduction in the frequency of diplopia in the primary position or maintenance of the deviation angle at ≤50% of the pre-injection angle at more than 6 months of follow-up after the last injection.
- No change: No change in diplopia or angle of deviation compared to pre-injection at more than 6 months of follow-up after the last injection.
- Repeated injections: Less than 6 months have passed since the last injection at the time of the final observation.
- Surgery: Esotropia surgery performed after injection.

### Adverse event definitions

Adverse events were evaluated based on established criteria from previous reports on botulinum toxin injection for strabismus [21–23], and defined as follows:

- Ptosis: The upper eyelid margin covering the pupillary margin on slit-lamp examination.
- Hypertropia: A newly developed vertical deviation not observed before the injection.
  o Overcorrection: A change from esotropia to exotropia exceeding 10 PD after BTXA injection.
  o Ocular motility dysfunction: Newly observed limitation of ocular movement on duction testing.

Although overcorrection and ocular motility dysfunction are not strictly classified as adverse events, they represent excessive pharmacologic effects of BTXA. However, because these events can lead to patient discomfort, they were also recorded. Additionally, since overcorrection did not necessarily accompany ocular motility limitation, the two were recorded separately.

### Statistical analysis

Categorical data are summarized as frequencies and percentages, whereas continuous data are expressed as mean ± standard deviation or median and range (minimum–maximum). A paired t-test was used to evaluate the changes in the distance angle of deviation from pre-treatment to 1-month post-injection, as well as from pre-treatment to the final observation in cases with a follow-up period of more than 6 months. The McNemar test was used to evaluate the changes in the proportion of distance esophoria and near stereopsis <100 arc seconds (arcsec) from pre-treatment to 1-month post-injection, as well as from pre-treatment to the final observation for cases with a follow-up period exceeding 6 months. An independent two-sample t-test was used to compare the BTXA dose between the group that experienced adverse events (blepharoptosis or hypertropia) at least once and the group that did not. A multivariable logistic regression analysis was performed to identify the factors contributing to the success of BTXA treatment. Based on the simple regression analysis results and clinically important factors, six explanatory variables were selected: duration from onset to treatment, dosage, pre-treatment angle of deviation, pre-treatment near stereopsis, post-treatment overcorrection, and post-treatment ocular motility dysfunction. Statistical analyses were performed using SAS version 9.4 (SAS Institute Inc.). All P-values were two-sided. Because this study was exploratory in nature, no adjustments were made to multiple comparisons.

## Results

### Patient demographics

The study included 228 patients diagnosed with AACE who received their first BTXA injection at the CS Eye Clinic from the same surgeon. The patient demographic characteristics are presented in Table 1.

**Table 1.** Demographic and clinical characteristics of included patients

Demographics	All (n = 228)
Age (years)	
Mean ± SD	30.9 ± 11.9
Median (min–max)	28 (12–75)
Sex (male), n (%)	93 (41%)
Duration from onset to treatment (months)	
Mean ± SD	54.8 ± 163.4
Median (min–max)	36.5 (0.2–486.7)
Distant Angle of Deviation (PD)	
Mean ± SD	28.9 ± 14.2
Median (min–max)	25 (4–95)
Distant Qualitative Eye Position, n (%)	
Esotropia	222 (97%)
Esophoria	6 (3%)
Near Stereopsis, n (%)	
Absent	76 (33%)
Present	152 (67%)
Observation Period (months)	
Mean ± SD	9.1 ± 8.2
Median (min–max)	6.7 (0.0–36.4)

*SD* standard deviation, *PD* prism diopter

The patients’ ages ranged from 12 to 75 years, with a mean age of 30.9 ± 11.9 years. The minimum age of 12 years was selected because, in Japan, public health insurance covers BTXA treatment for strabismus only for patients aged ≥12 years. The study population comprised 93 (41%) men and 135 (59%) women. The duration from symptom onset to the initiation of BTXA treatment varied significantly, with a median time to treatment of 36.5 months (range: 0.2–486.7 months). The mean angle of esotropia at a distance was 28.9 ± 14.2 PD. Most (97%) patients exhibited stable esotropia at a distance, while a small proportion (3%) had esophoria at a distance. Near stereopsis was absent and present in 76 (33%) and 152 (67%) patients, respectively. The mean observation period was 9.1 ± 8.2 months.

### Injection details

Overall, 318 BTXA injections were administered, with an average of 1.4 ± 0.8 injections per patient. The mean dosage per injection was 2.7 ± 0.9 units.

### Treatment outcomes

**Fig. 1:**
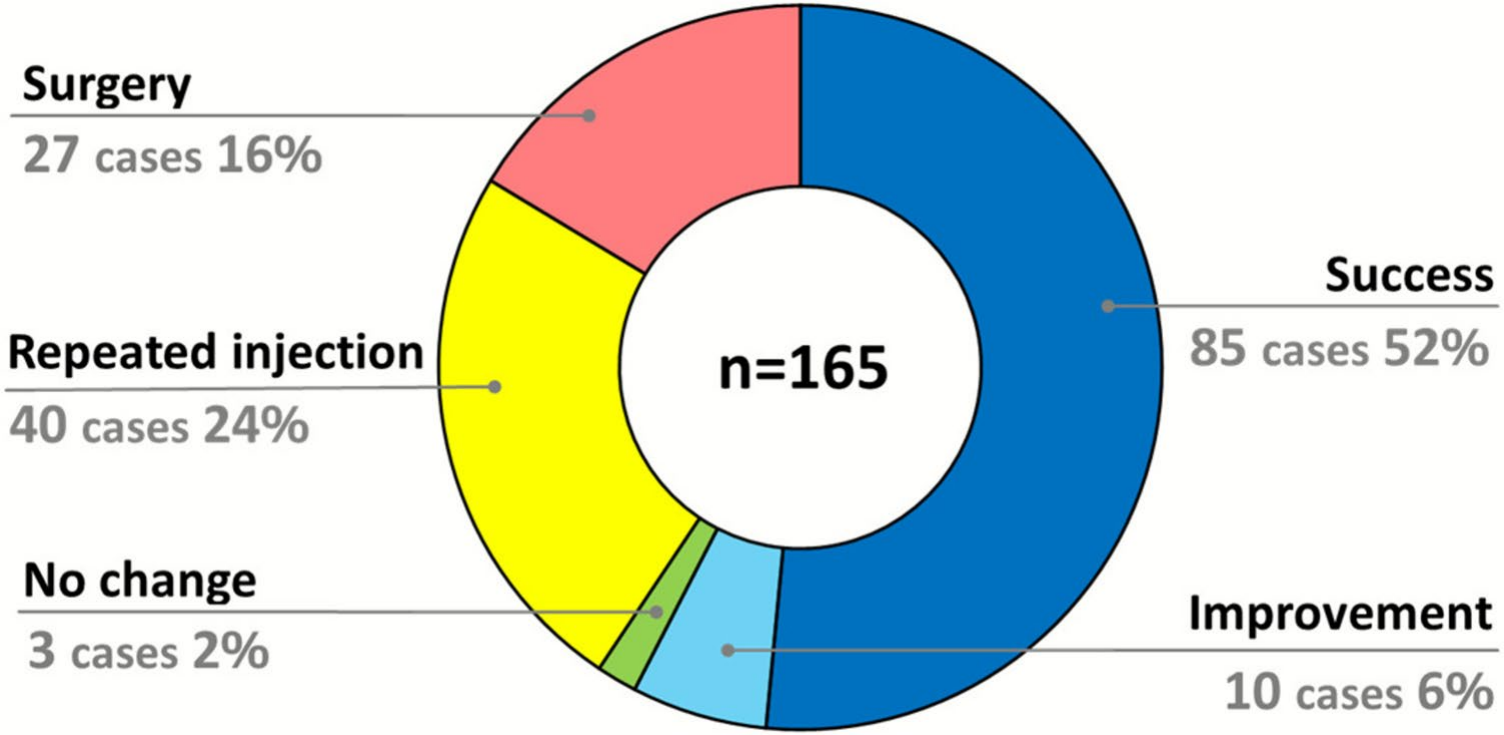
Treatment outcomes. Success was achieved in 85 (52%) patients, improvement in 10 (6%), and no change in 3 (2%). Repeated injections were required in 40 (24%) patients. Surgery was performed in 27 (16%) patients who did not opt for additional botulinum toxin type A (BTXA) injections due to recurrence, insufficient effect, or adverse events

The treatment outcomes of the 165 patients observed for more than 6 months after the initial injection are shown in Figure 1. Successful results were achieved in 85 cases (52%). Ten (6%) cases demonstrated improvement, and three (2%) showed no change.

A subset of 40 (24%) patients required repeated injections because of esotropia recurrence. Surgery was performed in 27 (16%) patients who did not opt for additional BTXA injections owing to recurrence, inadequate response, or adverse events.

### Changes in the distance angle of deviation

**Fig. 2:**
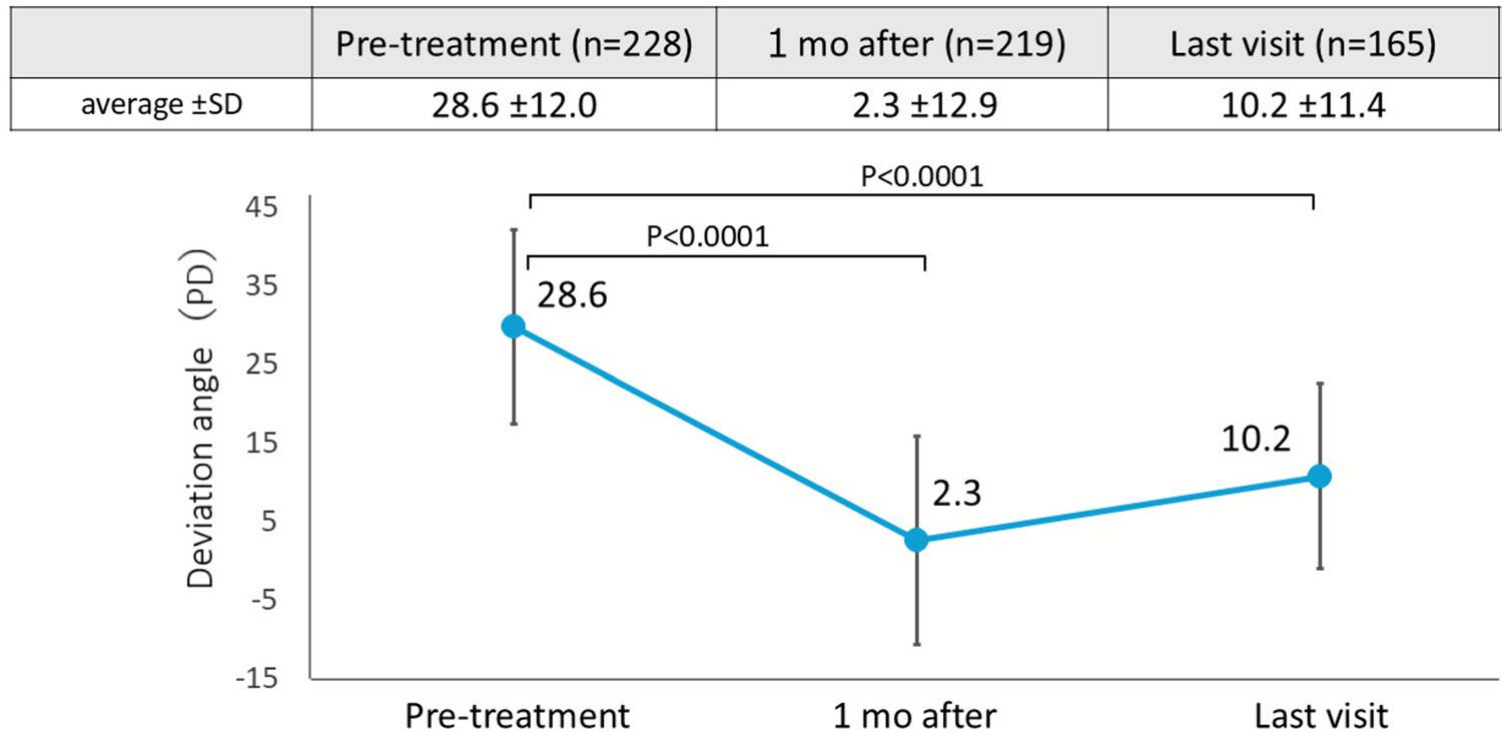
Longitudinal changes in the distance angle of deviation. The mean pre-treatment angle of deviation was 28.6 ± 12.0 prism diopter (PD). One month after treatment, it significantly decreased to 2.3 ± 12.9 PD (P < 0.0001). At the final observation, the angle of deviation slightly increased to 10.2 ± 11.4 PD, showing significant improvement compared to the pre-treatment angle (P<0.0001)

The changes in the distance angle of deviation over time are shown in Figure 2. Before the treatment, the mean angle of deviation was 28.6 ± 12.0 PD. One month after treatment, this angle significantly reduced to 2.3 ± 12.9 PD (P < 0.0001). At the final observation, the mean angle of deviation slightly increased to 10.2 ± 11.4 PD, indicating some degree of regression but still showing a marked improvement from the pre-treatment measurements (P<0.0001).

### Proportion of distance esophoria

**Fig. 3:**
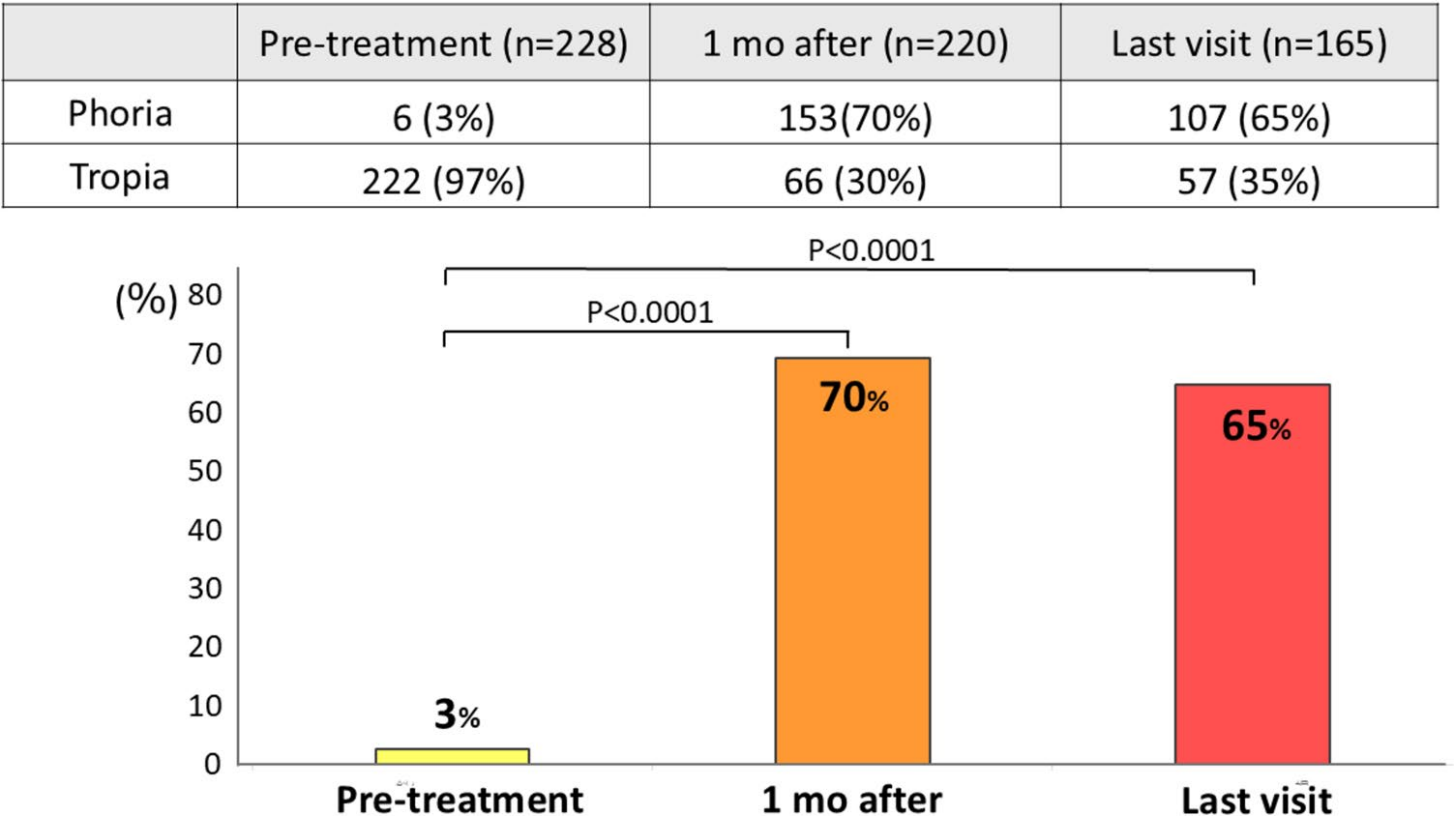
Proportion of distant esophoria. Before treatment, six (3%) patients had distant esophoria. One month after treatment, the number of patients with distant esophoria increased to 153 (70%) (P<0.0001). At the final observation, 107 (65%) patients had distant esophoria, showing a significant increase in the proportion of distant esophoria compared to pre-treatment (P < 0.0001).

Changes in the proportion of distance esophoria are shown in Figure 3. Before treatment, 222 (97%) patients had distance esotropia, while six (3%) patients had distance esophoria. One month after treatment, the number of patients with distance esotropia had decreased to 66 (30%), whereas that of those with distance esophoria had increased to 153 (70%) (P<0.0001). At the final observation, 57 (35%) patients had distance esotropia, and 107 (65%) patients had distance esophoria, showing a significant increase in the proportion of distance esophoria compared with pre-treatment (P<0.0001).

### Proportion of near stereopsis less than 100 arcsec

**Fig. 4:**
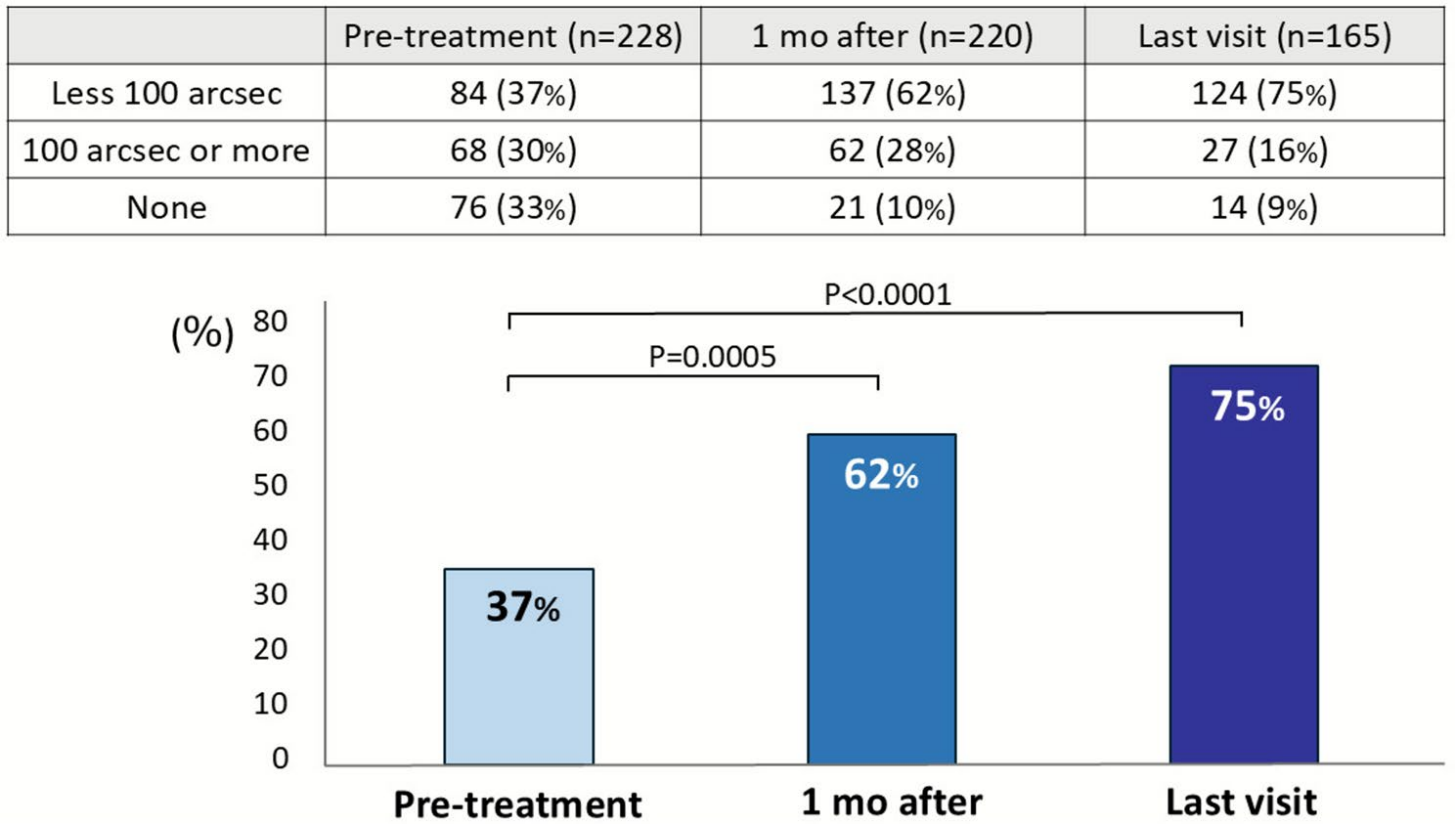
Proportion of near stereopsis <100 arcsec. Before treatment, 84 (37%) patients had near stereopsis <100 arcsec. One month after the treatment, 137 (62%) patients achieved near stereopsis <100 arcsec. At the final observation, 124 (75%) patients had near stereopsis <100 arcsec, demonstrating a significant increase in the proportion of patients with good near stereopsis over time (at 1 month, P=0.0005; at the final observation, P<0.0001).

The proportion of patients with good near stereopsis <100 arcsec also showed notable changes (Fig. 4). Before treatment, 84 (37%), 68 (30%), and 76 (33%) patients had near stereopsis <100 arcsec, near stereopsis ≥100 arcsec, and no stereopsis, respectively. One month after treatment, 137 (62%), 62 (28%), and 21 (10%) patients achieved near stereopsis <100 arcsec, near stereopsis ≥100 arcsec, and no stereopsis, respectively. At the final observation, 124 (75%), 27 (16%), and 14 (9%) patients had near stereopsis <100 arcsec, near stereopsis ≥100 arcsec, and no stereopsis, respectively, indicating a significant improvement in near stereopsis over time. The proportion of patients with good near stereopsis significantly increased over time (1 month, P=0.0005; final observation, P<0.0001).

### Adverse events

Adverse events were observed in 224 patients, as summarized in Table 2. The most common adverse event was blepharoptosis, which occurred in 53 (24%) patients, followed by hypertropia in 35 (16%). Overcorrection and ocular motility dysfunction, which were considered excessive pharmacologic effects rather than true adverse events, were observed in 49 (22%) and 33 (15%) patients, respectively. All of these events were transient and resolved spontaneously within approximately 1 month. In some cases, multiple events occurred in the same patient.

**Table 2.** Adverse events

Adverse Event	Number of Cases (%) n = 224
Blepharoptosis	53 (24%)
Hypertropia	35 (16%)
Overcorrection	49 (22%)
Ocular Motility Dysfunction	33 (15%)

Blepharoptosis was observed in 24% of patients, and hypertropia was observed in 16%. Although overcorrection and ocular motility dysfunction are the intended effects of the drug rather than adverse events, they are included in the same table, as they cause discomfort to the patients

### Relationship between dosage and adverse events

The relationship between the dosage of BTXA and the occurrence of adverse events was examined. The group that experienced adverse events, including blepharoptosis or hypertropia (n=78), received a higher average dose of BTXA than the group without these adverse events (n=146). The average dose in the adverse event group was 3.01 ± 0.99 units, while the group without adverse events received 2.60 ± 0.87 units; this difference was statistically significant (P =0.001).

### Factors contributing to success

The analysis suggests that the duration from onset to treatment (P=0.018) and post-treatment overcorrection (P=0.0073) independently influenced the likelihood of success. Specifically, a shorter duration from onset to treatment and post-treatment overcorrection were associated with higher success rates. The results are summarized in Table 3.

**Table 3.** Factors Contributing to Success (Multivariable Logistic Regression Analysis)

Explanatory Variables	Odds Ratio (95% CI)	*p*-value
Duration from Onset to Treatment	0.992 (0.986–0.999)	0.018*
Dosage	0.831 (0.529–1.310)	0.42
Pre-treatment Angle of deviation	1.020 (0.978–1.060)	0.391
Pre-treatment Near Stereopsis	2.060 (0.945–4.470)	0.0693
Post-treatment Overcorrection	3.550 (1.410–8.970)	0.0073*
Post-treatment Ocular Motility Dysfunction	1.980 (0.640–6.150)	0.235

The analysis suggested that the duration from onset to treatment (*P*=0.018) and post-treatment overcorrection (*P*=0.0073) independently influence the likelihood of success. CI, confidence interval

## Discussion

This study evaluated the clinical efficacy, safety, and success factors associated with BTXA injections in treating AACE. The results revealed significant improvements in eye alignment and stereopsis with BTXA treatment and identified success factors contributing to maintaining long-term favorable outcomes beyond the active period of the drug. Although transient adverse events were observed in approximately 20% of the cases, all were temporary and resolved within approximately 1 month. Higher doses of BTXA are associated with an increased incidence of blepharoptosis and hypertropia. Multivariable logistic regression analysis revealed that a shorter time from onset to treatment and post-treatment overcorrection were independent success factors. This finding strongly suggests that early intervention is crucial for achieving success. However, overcorrection can be uncomfortable for patients, making it impractical as an initial goal. A clinically appropriate strategy would involve evaluating the response following the initial injection and adjusting the dosage to accommodate each patient accordingly.

This study represents the largest series of 228 cases involving 318 injections performed by the same surgeon at a single institution to evaluate the outcomes of BTXA injections for strabismus. This and previous studies [12, 13, 15, 24] show favorable outcomes with BTXA injections for AACE. With the increasing use of digital devices, the incidence of AACE is also rising and is becoming a growing concern [2, 6, 8, 10, 16, 24–27]. Unlike surgery, which cannot be performed until the condition stabilizes, BTXA injections can be administered during the acute phase, making them particularly effective in cases of acquired strabismus, such as AACE, where patients complain of diplopia. Additionally, BTXA injections are less invasive than surgery, can be administered quickly during an outpatient visit, and cause minimal subsequent discomfort. Therefore, BTXA injections should be considered a viable AACE treatment option.

The incidence of complications, including blepharoptosis and hypertropia, after the injections was consistent with that of previous reports [28, 29]. Although complications occurred at a certain rate, none were permanent, indicating that BTXA injections are a safe treatment option. This study also suggests that a higher dose is associated with an increased likelihood of complications. Therefore, a more careful pre-treatment explanation is necessary for cases with larger esotropia angles that require higher dosages. One potential approach to reduce complication rates is to inject into both medial rectus muscles, thereby reducing the dosage per muscle. In recent cases, when a total dose of 4.0 units or more is required, we usually divide the dose between the two medial rectus muscles. We have clinically observed that this approach achieves a comparable degree of horizontal correction to that obtained when the entire dose is injected into a single eye, while reducing the risk of adverse events.

Although the effects of BTXA typically diminish within 3–4 months, some reports suggest that BTXA injections can cause irreversible changes in muscle fibers, particularly in delicate extraocular muscles [30]. This study revealed that approximately 57% of the patients who had received BTXA injections maintained good ocular alignment without diplopia for more than 6 months post-injection. AACE frequently occurs in patients who initially have good binocular function and controlled phoria but develop esotropia and notice diplopia at a distance after engaging in excessive near work. Using BTXA injections to induce phoria and adopting lifestyle habits, including maintaining proper viewing distance from digital devices, limiting screen time, and regularly focusing on distant objects, may help restore the patient’s ocular alignment to its pre-esotropia state without the need for surgery.

This study had some limitations. First, the mean follow-up period was relatively short (approximately 9 months). Therefore, uncertainty remains regarding the long-term stability of ocular alignment after BTXA injection, and caution is warranted to avoid overinterpreting the findings. Previous studies comparing BTXA and surgery for AACE show inconsistent results; while some reports suggest that surgery provides more precise and durable long-term alignment [24], other studies demonstrate that BTXA can yield non-inferior outcomes compared with surgery at 36 months [31]. Future studies with extended follow-up periods are needed to more clearly evaluate the long-term effects of BTXA. Second, this study had a high dropout rate (63 of the 228 cases were lost to follow-up 6 months after the initial injection), and its retrospective nature may introduce potential selection bias. Additionally, the data for this study were derived from a single institution. To better understand the dropout pattern, we compared baseline characteristics between the dropout group (n = 63) and the total cohort (n = 228), including age, sex, duration from onset to treatment, and presence of diplopia. The dropout group tended to include a higher proportion of patients in their twenties (50.8% vs. 37.7% overall) and a greater proportion without diplopia (15.9% vs. 9.0%), whereas the sex ratio and time from onset to treatment were similar between the two groups. These results suggest two possible reasons for the relatively high dropout rate: (1) younger patients in their twenties had lower follow-up compliance and discontinued visits after their symptoms improved following BTXA injection, and (2) some patients without diplopia did not feel the need for follow-up visits because they experienced no difficulties in daily life. However, given its large sample size, this study likely plays a meaningful role in assessing the efficacy and safety of BTXA injections for strabismus. Future multi-center, prospective studies are required to further verify the efficacy of BTXA for AACE.
